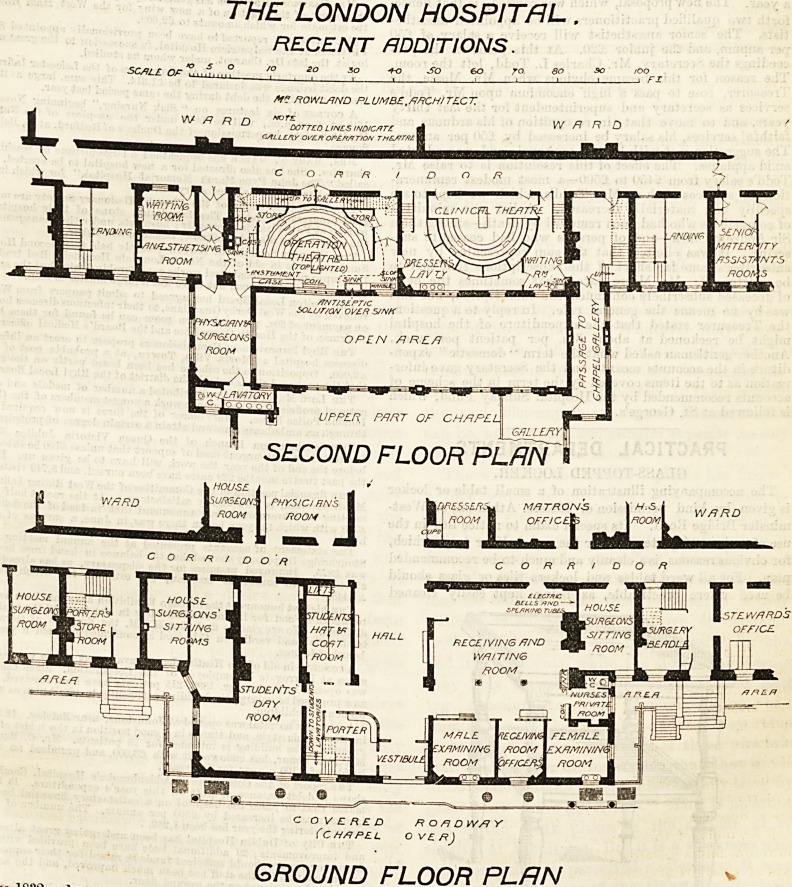# The London Hospital New Buildings

**Published:** 1893-11-18

**Authors:** 


### HOSPITAL CONSTRUCTION.

### THE LONDON HOSPITAL NEW BUILDINGS.

We publish to-day the plans of some extensive and much-needed improvements at the London Hospital recently completed under the direction of the architect, Mr. Rowland Plumbe, F.R.I.B.A. All who knew the hospital before the recent alterations will remember the long recessed front of the building towards the Whitechapel Road, with the Grocers' wing flanking it on one side, and the House Governor's house on the other. In the centre was the main entrance, approached by a flight of steps, up which patients had to be carried in the open air and in full view of the public road. Inside, the corridors of communication from east to west were blocked on the first and second floors by the chapel, and as a consequence the wards became the passages in ordinary use. The operation theatre and its appurtenances were wholly inadequate to the needs of this great hospital with its large medical school, and the need of a theatre for clinical teaching was also strongly felt. The new buildings have been devised to meet these requirements, and are certainly well adapted to the objects the authorities had in view. On the ground floor is a spacious covered roadway, with an entrance and exit to the road, and rising with an easy gradient to within two steps of the ground floor level of the hospital. On the north or Whitechapel Road side of this roadway is a covered space to serve as shelter for the friends of patients, who frequently come long distances and have to wait in all weathers until the visiting hour arrives. A lodge and a room for hand ambulances are also placed on this side of the roadway. On the left of the main entrance is the porter's lodge, and a large day room and a cloak room for students. A staircase close to these rooms leads to students' lavatories in the basement. On the left of the entrance hall is a large receiving and waiting hall for patients, a small room for a nurse, rooms for examining male and female patients, and the receiving officer's room. These rooms are all lighted by sky-lights from the open area which will be seen on the first floor plan between the chapel and the main building. On the first floor is the chapel, which is built over the covered roadway, and the space it formerly occupied has been converted into a continuation of the main corridor and rooms for the resident staff. At the chancel end of the chapel is a room for the chaplain, with w.c. and entrance to the chapel. On the second floor the corridor is again continued through where the upper part of the chapel formerly cut off communication. Here have been formed two large theatres—one for operations, the other for clinical teaching. The operation theatre is lined throughout with glazed tiles, and has a floor of marble mosaic. It is lighted by a large horizontal light in the ceiling, over which is a glass roof, and by a large vertical window facing north. The access for students to the gallery is by way of two staircases leading directly from the corridor. Against the wall at the back and to the left hand of the operator is the instrument case; to the right is a large sink, over which are taps and pipes connected with tanks for disinfectants placed in the gallery. Next to this is a marble slab, on which is a small wringer for drying sponges, &c., and in the corner is a slop shoot. A door close by leads into the space under the seats in the clinical theatre, where are placed three lavatory basins for dressers. In the space under the gallery on the left of the theatre are arranged cases for dressings, &c., and a sink and table for the instrument man. Adjoining the theatre is a room for the administration of anæsthetics, and a room for the surgeons with lavatory and w.c. attached. On the third floor some additional rooms for nurses and servants have been arranged, and in the basement besides stores, lavatories, &c., there is a new set of baths for out-patients. In addition to the works described above, the whole of the drainage and sanitary arrangements have been reorganised. For this part of the work the Governors called in the aid of Dr. Louis Parkes, M.D., D.P.H., the Assistant Professor of Hygiene at University College, under whose advice, acting in conjunction with Mr. Plumbe, the much needed improvements have been carried out. These works comprise the removal, so far as was consistent with the safety of the buildings, of all the brick sewers and drains, some of which had been in existence since 1832 and were disused, and of all contaminated earth around them. An entirely new system of drainage formed of tested stoneware pipes with all necessary disconnection traps, manholes, &c., and new sanitary towers for w.c.'s, sinks, &c., with disconnecting lobbies to the east and west wings and to the Alexandra wards. The existing sanitary fittings, such as slop-sinks and the like, were replaced with others of an improved type, and all soil and waste pipes removed from the inside of the building and replaced by new pipes outside and properly ventilated.

### THE LONDON HOSPITAL.

### RECENT ADDITIONS.

### GROUND FLOOR PLAN

## Figures and Tables

**Figure f1:**